# Time-Interleaved SAR ADC with Background Timing-Skew Calibration for UWB Wireless Communication in IoT Systems

**DOI:** 10.3390/s20082430

**Published:** 2020-04-24

**Authors:** Kiho Seong, Dong-Kyu Jung, Dong-Hyun Yoon, Jae-Soub Han, Ju-Eon Kim, Tony Tae-Hyoung Kim, Woojoo Lee, Kwang-Hyun Baek

**Affiliations:** 1 School of Electrical and Electronics Engineering, Chung-Ang University, Seoul 06974, Korea; tjdrlgh@cau.ac.kr (K.S.); jdgring@cau.ac.kr (D.-K.J.); kindyoon@cau.ac.kr (D.-H.Y.); lynn2776@cau.ac.kr (J.-S.H.); space@cau.ac.kr (W.L.); 2 School of Electrical and Electronics Engineering, Nanyang Technology University, Singapore 639798, Singapore; jueon.kim@ntu.edu.sg (J.-E.K.); THKIM@ntu.edu.sg (T.T.-H.K.)

**Keywords:** analog-to-digital converter (ADC), successive approximation register (SAR) ADC, time-interleaved SAR ADC, timing-skew calibration, comparator offset, ultra-wideband (UWB) wireless communication

## Abstract

Ultra-wideband (UWB) wireless communication is prospering as a powerful partner of the Internet-of-things (IoT). Due to the ongoing development of UWB wireless communications, the demand for high-speed and medium resolution analog-to-digital converters (ADCs) continues to grow. The successive approximation register (SAR) ADCs are the most powerful candidate to meet these demands, attracting both industries and academia. In particular, recent time-interleaved SAR ADCs show that multi-giga sample per second (GS/s) can be achieved by overcoming the challenges of high-speed implementation of existing SAR ADCs. However, there are still critical issues that need to be addressed before the time-interleaved SAR ADCs can be applied in real commercial applications. The most well-known problem is that the time-interleaved SAR ADC architecture requires multiple sub-ADCs, and the mismatches between these sub-ADCs can significantly degrade overall ADC performance. And one of the most difficult mismatches to solve is the sampling timing skew. Recently, research to solve this timing-skew problem has been intensively studied. In this paper, we focus on the cutting-edge timing-skew calibration technique using a window detector. Based on the pros and cons analysis of the existing techniques, we come up with an idea that increases the benefits of the window detector-based timing-skew calibration techniques and minimizes the power and area overheads. Finally, through the continuous development of this idea, we propose a timing-skew calibration technique using a comparator offset-based window detector. To demonstrate the effectiveness of the proposed technique, intensive works were performed, including the design of a 7-bit, 2.5 GS/s 5-channel time-interleaved SAR ADC and various simulations, and the results prove excellent efficacy of signal-to-noise and distortion ratio (SNDR) and spurious-free dynamic range (SFDR) of 40.79 dB and 48.97 dB at Nyquist frequency, respectively, while the proposed window detector occupies only 6.5% of the total active area, and consumes 11% of the total power.

## 1. Introduction

The fourth industrial revolution is upon us. Internet-of-things (IoT), big data analytics, and artificial intelligence (AI) are the representative leading-edge technologies that serve as enablers and facilitators of this revolution. In particular, the explosive growth of data produced via IoT has been playing a pivotal role in building big data and creating AI learning data. The proliferation of the IoT is, in turn, attributable to tremendous researches and engineering efforts to develop new methodologies and techniques for low-power designs and wireless communication systems [1,2,3,4].

Ultra-wideband (UWB) wireless communication is one of the contributors to the prosperity of the IoT and is also one of the representative beneficiaries that is continuing to evolve with the IoT. With the continued development of UWB wireless communications, the demand for multi-GS/s (giga sample per second) high-speed and medium-resolution analog-to-digital converters (ADCs) have been ever-increasing [5,6,7,8,9,10]. To meet the high-speed requirements, flash ADCs have been the most widely used in UWB applications. However, these ADCs have decisive limitations in terms of power consumption and area as ADC resolution increases. Because of the limitations, most flash ADCs have been designed with less than 6-bit resolution [11,12,13,14], which makes it difficult to meet today’s medium-resolution requirements.

The successive approximation register (SAR) ADCs are well-known power and area-efficient ADCs. In addition, the digital affinity of SAR ADCs enables scalability to various semiconductor technologies, thereby accelerating versatility. However, the SAR ADCs are not as fast as the flash ADCs because of their unique operating characteristic. Even if the SAR ADCs are designed to be relatively high speed, the power efficiency tends to be faded because power-hungry comparators and fast capacitive-digital-to-analog converter (CDAC) settling time are required.

To enable high-speed ADC designs while maintaining the power-efficient advantage, researches to apply the time-interleaved architecture to the SAR ADC have been intensively studied [5,15,16,17,18,19,20,21,22,23]. Parallel operations of several slow-running sub-ADCs provide the same parallelization effect as a single fast ADC, enabling the low-power and high-speed ADCs. Unfortunately, these time-interleaved SAR ADCs still have a critical drawback; mismatches between the sub-ADCs can significantly degrade overall ADC performance. More specifically, the time-interleaved ADCs suffer from offset mismatch, gain mismatch, and sampling timing skew between channels. Fortunately, the offset and gain mismatches cause constant errors regardless of the input frequency, so they can be easily corrected in the digital domain. However, calibrating the sampling timing skew is very challenging, because the errors strongly depend on the input frequencies [24].

**Figure 1:**
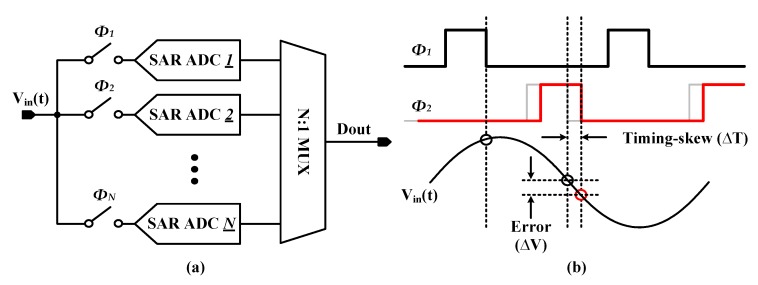
(**a**) Basic structure of a time-interleaved successive approximation register analog-to-digital converters (SAR ADC) and (**b**) timing-skew error in a time-interleaved SAR ADC.

The sampling error induced by the (sampling) timing skew can be analyzed in Figure 1. Figure 1a shows the basic structure of a time-interleaved SAR ADC that consists of *N* sub-ADCs. Given that the input *V_in_(t)* is applied to the sub-ADCs, as seen in Figure 1b, the timing skew (∆*T*) gives rise to the voltage error (∆*V*). The precise relationship between ∆*T* and ∆*V* is as follows:

(1) $$\Delta V\approx \frac{\partial Vin(t)}{\partial t}\cdot \Delta T$$

Meanwhile, for a sinusoidal input, the signal-to-noise ratio (SNR) of the time-interleaved ADC, which includes the quantization noise and the timing-skew error, can be expressed as:

(2) $$SNR=\frac{1}{\frac{1}{6}{\left(\frac{2}{2^{M}}\right)}^{2}+\pi^{2}\Delta T^{2}{f_{in}}^{2}}$$

where *M* and *f_in_* represent the ADC resolution and the input frequency, respectively. From the above equations, it can be seen that the timing skew (∆*T*) degrades the SNR significantly as *f_in_* increases. 

**Figure 2:**
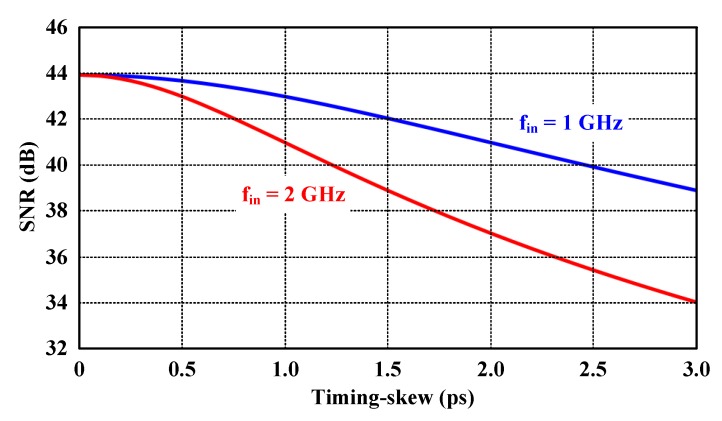
Signal-to-noise ratio (SNR) degradation of the 7-bit time-interleaved ADC due to the timing skews.

The MATLAB simulation results of the 7-bit time-interleaved ADC reported in Figure 2 show a more intuitive relationship between the timing skew and the SNR degradation. As seen in the figure, faster *f_in_* causes greater SNR degradation. To achieve the SNR above 40 dB, for example, the timing-skew error must be lower than 2.5 ps and 1.2 ps in case *f_in_* = 1 GHz and *f_in_* = 2 GHz, respectively.

There have been extensive studies to overcome the timing-skew error problem of the time-interleaved ADCs, and the various timing-skew calibration techniques have been proposed [14,15,16,18,19,20,21,22,23,25,26,27,28,29,30]. In Section 2, the previous timing-skew calibration techniques will be reviewed in detail. Then, Section 3 introduces the proposed timing-skew calibration technique using a comparator offset-based window detector. The proposed technique includes a calibration algorithm that comes with a window detector design that is area efficient and robust to process–voltage–temperature (PVT) variations. More precisely, the detailed design and operating principle of the proposed window detector are presented in Section 3.1, while the proposed timing-skew calibration algorithm is elucidated in Section 3.2. Section 4 is dedicated to presenting detailed information about the simulation results, and Section 5 concludes the paper.

## 2. Timing-Skew Calibration Techniques: A Review

One of the best-known timing-skew calibration techniques is the statistic-based scheme proposed in several previous literatures [14,18,25,27,29]. This scheme tries to resolve the timing-skew error by maximizing the correlation between sub-ADCs or between sub-ADC and reference ADC. Due to the assumption that the input statistics are wide-sense-stationary (WSS), the statistic-based scheme has serious constraints on the input. In other words, if the input characteristics, such as frequency and statistics, change frequently, the WSS will be invalid and will limit the calibration, significantly reducing calibration accuracy. 

Meanwhile, the derivative-based timing-skew calibration scheme was proposed in [21,26]. This scheme exploits an auxiliary ADC to get the input derivative and then calibrates for it by extracting the direction and magnitude of the timing skew. Although this scheme effectively reduces timing-skew errors, it has a disadvantage of very high design complexity. Furthermore, this scheme is vulnerable to the PVT variations because it delays the input in the analog domain.

In addition, the flash-assisted timing-skew calibration scheme was introduced in [16,28]. This scheme adopts a flash-ADC as a timing-skew estimator and achieves considerable error mitigation. However, the power-consuming flash ADC results in power-efficiency degradation. Moreover, as the sampling speed increases, the power consumption may grow exponentially. 

**Figure 3:**
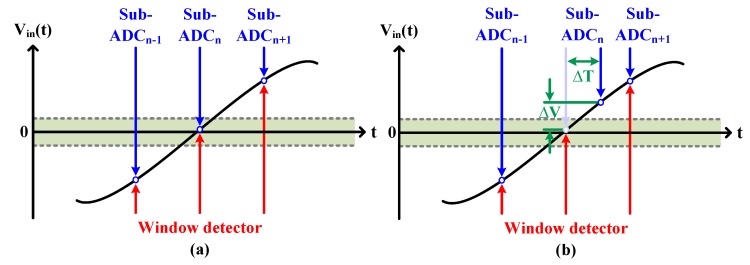
Concept of the window detector-based timing-skew calibration (**a**) without timing skew (**b**) with timing skew.

More recently, a timing-skew calibration scheme using a window detector was proposed [20,22,23]. The basic motivation for this scheme is that the timing-skew information can be obtained by detecting whether ∆*V* is within a specified window region or not. For example, when the window detector determines that the input is within the window, one of the sub-ADCs samples the input inside the window if there is no timing skew. This case is described in Figure 3a; the sub-ADC_n_ samples the input within the window. On the other hand, if the timing-skew exists, the corresponding sub-ADC_n_ will sample the input outside the window, which is illustrated in Figure 3b. Eventually, the timing skew can be calibrated by adjusting the sampling clock, thus allowing ∆*V* to enter the window region.

**Figure 4:**
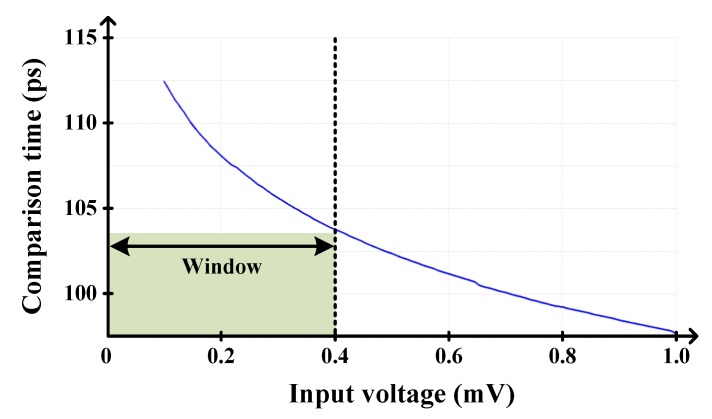
Relationship between the comparison time and input voltage.

Then, detecting whether the input is in the specific window region or not is critical in this scheme. To this end, a detection technique using a comparator and its comparison time was presented in [20,23]. As shown in Figure 4, if the input voltage falls into the window region, the comparison time becomes longer than a certain time. Based on this relationship, a specific delay can be set and used to determine whether the input is in the window or not. Despite the successful detectability of this technique, additional calibration logics are required because the delay cell and comparison time are vulnerable to the PVT variations. And, unfortunately, the additional logics inevitably increase design complexity and power consumption.

**Figure 5:**
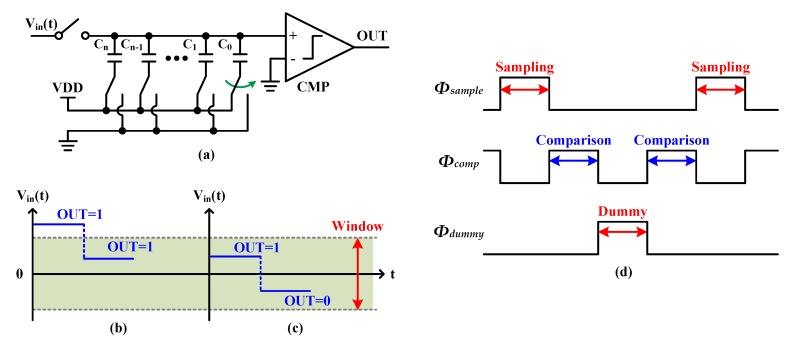
(**a**) Basic structure of the SAR-based window detector and its operating principle when (**b**) outside the window and (**c**) inside the window. The timing diagram is shown in (**d**).

To offload the additional calibration logics, a SAR-based window detector has recently been proposed [22]. Figure 5a shows the basic structure of the SAR-based window detector, and Figure 5b and c show the concept of its operating principle which consists of two steps: The first step is to determine whether the input voltage is greater or less than zero (which is the most significant bit (MSB) of the SAR conversion), and the second step is to switch the CDAC and then determine whether it is greater than zero again. If the input voltage is out of the window region, the comparator outputs of the first and second steps should be the same (Figure 5b). Otherwise, the two outputs should be different from each other (Figure 5c). Note that because the SAR-based window detector requires at least two comparison cycles (*Φ_comp_*), the clock cycle following the sampling clock (*Φ_sample_*) is used for comparison rather than sampling, as shown in Figure 5d. Therefore, the input impedance varies, and eventually, it causes the bandwidth mismatch. To solve this bandwidth mismatch problem, an extra dummy-SAR ADC is included in [22] to sample the input alternately with *Φ_sample_* (*Φ_dummy_*). However, because of the extra dummy-SAR ADC, which occupies a large area, the presented technique still has critical limitations.

This paper presents a new window detection scheme and the timing-skew calibration algorithm for the high-speed time-interleaved SAR ADCs. The proposed window detector is more resistant to PVT variations, and it has lower digital complexity than previous works [20,23]. In addition, it has a higher area efficiency than [22].

## 3. Proposed Timing-Skew Calibration Scheme

**Figure 6:**
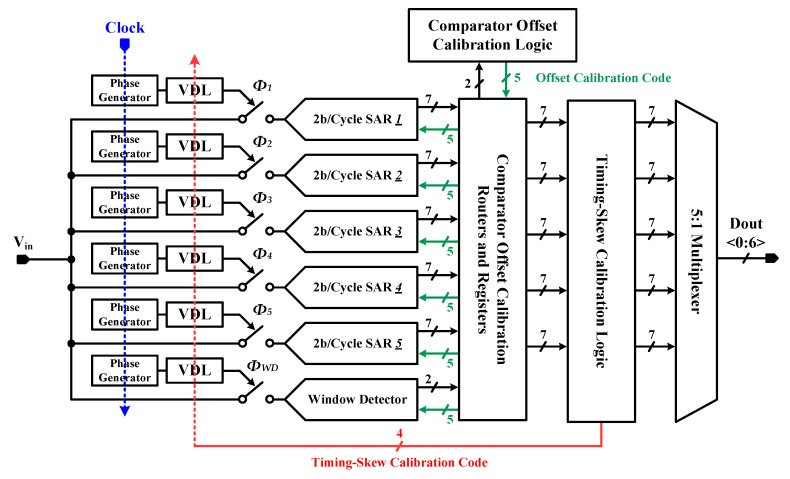
Block diagram of the proposed time-interleaved SAR ADC.

**Figure 7:**
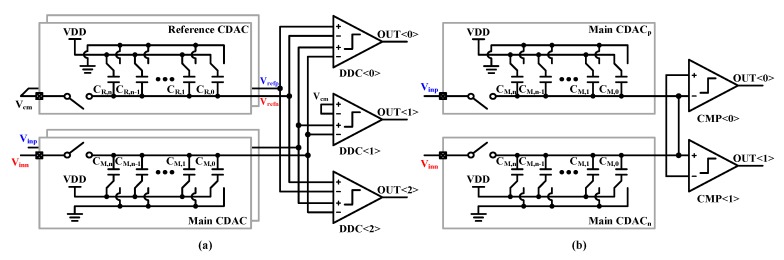
Block diagram of (**a**) the sub-channel 2b/cycle SAR ADC and (**b**) window detector SAR ADC.

The block diagram of the proposed time-interleaved SAR ADC is described in Figure 6. The sub-channel SAR ADC and window detector SAR ADC are illustrated in Figure 7a and b, respectively. 

The sub-ADCs basically utilized the 2b/cycle architecture presented in [31,32,33], whereby conversion speed can be significantly enhanced. As seen in Figure 7a, the three differential-difference comparators (DDCs) [34] compare the sampled input differential voltage with three reference voltages, which are generated by reference CDACs. And they resolve 2-bit in each decision cycle. The nonbinary decision scheme is adopted to compensate for CDAC settling error and kickback noise [31,35]. Eventually, ADC resolves 8-bit, including 1-bit redundancy, and then the nonbinary-to-binary decoder convert it to 7-bit binary codes.

The window detector SAR ADC has a similar structure to sub-ADCs, except for the input cross-coupled comparators, which are used for window detection. The detailed explanations are mentioned in the following subsections.

The phase generator generates sampling clock phases of the sub-ADCs (*Φ_1-5_*) and that of the window detector SAR ADC (*Φ_WD_*). Then the timing-skew calibration logics control the VDLs to align *Φ_1-5_* with *Φ_WD_*.

The single offset calibration logic not only compensates the sub-ADC’s offset but also makes the input cross-coupled comparators act as window detector. The total 17 comparators are used in the proposed architecture: 15 DDCs are used in the sub-ADCs, and the other two comparators (i.e., these are the differential comparators) are used in the window detector ADC. All these comparators are sequentially calibrated by single calibration logic and the calibration data are stored in the register in each ADC. 

### 3.1. Comparator Offset-based Window Detector

**Figure 8:**
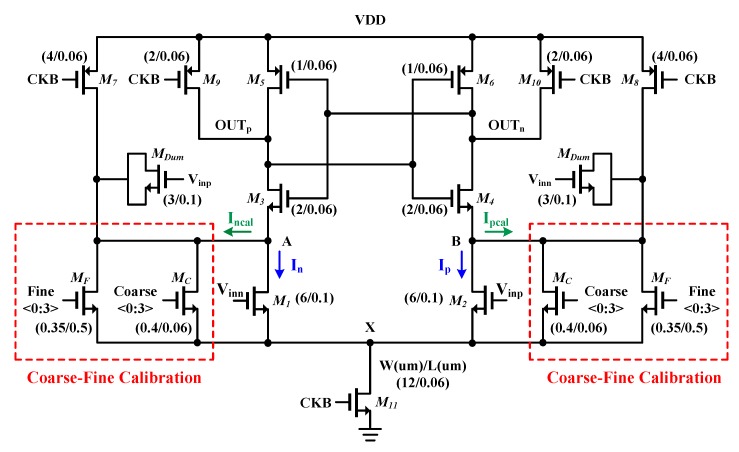
Schematic of the differential comparator with offset calibration transistors.

The proposed window-detector SAR ADC has two differential comparators implemented in the Strong-ARM latch-based dynamic comparators [36], so as to operate at high speed while maintaining high power efficiency. Figure 8 shows the detailed schematic of this differential comparator which consists of pre-amp (*M_1,2,11_*), latch (*M_3-6_*), reset switches (*M_7-10_*), dummy input transistors (*M_Dum_*) for kick-back noise reduction, and offset calibration transistors (*M_F,C_*).

Because the input difference of comparator makes different currents, if V_inp_ > V_inn_, then I_p_ > I_n_, it leads to OUT_p_ and OUT_n_ to be VDD ("1") and GND ("0"), respectively, due to the regeneration of the latch. (i.e., we define that the comparator output is "1" when OUT_p_ is VDD). However, if there is an offset, despite V_inp_ > V_inn_, it makes I_n_ > I_p_. In this case, the comparison error can be corrected by calibration current (I_pcal_) by turning on *M_F,C_* connected in parallel with input transistor *M_2_*.

If there is no offset, and the input difference is zero, the comparator outputs will be randomly distributed to "1" or "0". However, if there is an offset, the outputs will remain "1" or "0", even though the input difference is zero. Therefore, by applying the input difference to zero and monitoring the comparator outputs, it is always possible to see if there is an offset. And, from this confirmation, the offset calibration can be conducted.

**Figure 9:**
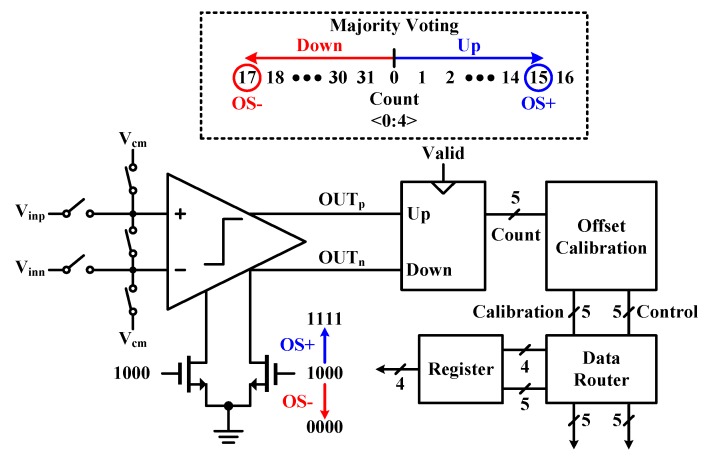
Block diagram of the offset calibration logic with the majority voting scheme.

Figure 9 presents the block diagram of the offset calibration logic. After shorting the inputs to the common-mode voltage (V_cm_), the comparator outputs control the up–down counter. If the comparator keeps outputs either "1" or "0", the up–down counter will reach the specific code (OS+ or OS-), and then it turns on or off the calibration transistors (*M_F,C_*).

If a small offset remains after a series of the offset corrections, the comparator output may appear slightly alternating between "1" and "0", it can be detected by majority voting scheme [37]. That is, the up–down counts compete and then, one of them reaches the specific code first, the calibration is performed.

**Figure 10:**
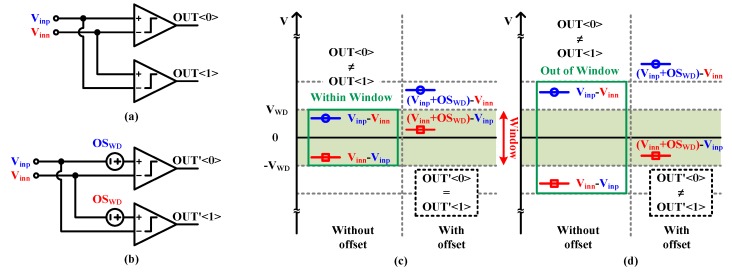
The input cross-coupled comparators (**a**) without and (**b**) with offset, and the comparator outputs equality (**c**) inside, and (**d**) outside window.

As aforementioned, we propose to use the comparators which have inevitable offset as the window detector. Figure 10a and b show the input cross-coupled comparators without and with offset (OS_WD_), respectively. As seen in Figure 10c and d, if there is no offset, the comparator outputs are must always be opposite, regardless of the input voltage. However, if there is an offset, the comparator outputs are the same when the input voltage falls into the window region. Whereas, if the input voltage is out of the window region, the comparator outputs are opposite again. Therefore, the input cross-coupled comparators with inevitable offset can be used as the window detector.

In this scheme, the smaller window width, the longer calibration convergence time, so it may not be able to keep up with temperature and voltage variations that have a significant effect on timing skew. On the other hand, the larger window width, the lower calibration accuracy. 

**Figure 11:**
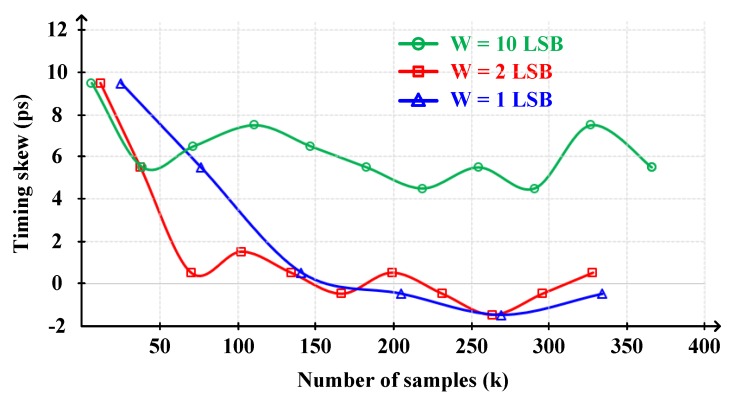
Relationship between window width, calibration accuracy and convergence time.

Figure 11 shows the relationship between window width, calibration accuracy, and convergence time. If W = 1 least significant bit (LSB), it needs more than 130 k samples to converge within ±1 ps skew. Whereas, if W = 2 LSB, it needs 70 k samples to converge within ±1 ps skew. Its convergence time is half of W = 1 LSB while maintaining ±1 ps accuracy. On the other hand, if W = 10 LSB, although it needs only 40 k samples to converge, its accuracy is very poor which is ~6 ps. Therefore, taking this trade-off between convergence time and accuracy into consideration, we determine to set the window width to 2 LSB (±1LSB, ±15 mV).

Since the window width of the proposed offset-based window detector is determined by offset, it is necessary to control the offset to set the window width. To force the desired offset, the unwanted offset induced from the process random mismatch must be first compensated.

To this end, it is important to know how much offset could be caused by process mismatch. From the Monte-Carlo simulation and post-layout parasitic extraction, the offset is estimated to be ±48 mV. The additional calibration transistors are connected in parallel with the input transistors to compensate the offset. To control the offset 1.5 mV accuracy, the compensation current (I_pcal_ and I_ncal_ in Figure 8) must be much smaller than the main current (I_p_ and I_n_ in Figure 8). To this end, long-channel transistors are used for calibration. Furthermore, to calibrate such ±48 mV offset, it is necessary that more than 6-bit extra transistors are needed. It results in large parasitic capacitance and limits the speed of the comparator. To tackle this issue, we adopt the coarse-fine calibration; that is, first the short-channel transistors cover ±48 mV range with 8.5 mV accuracy, then the long-channel transistors control the offset with 1.5 mV accuracy. Therefore, only 4-bit long-channel transistors are required, allowing accurate calibration while maintaining the comparator speed.

**Figure 12:**
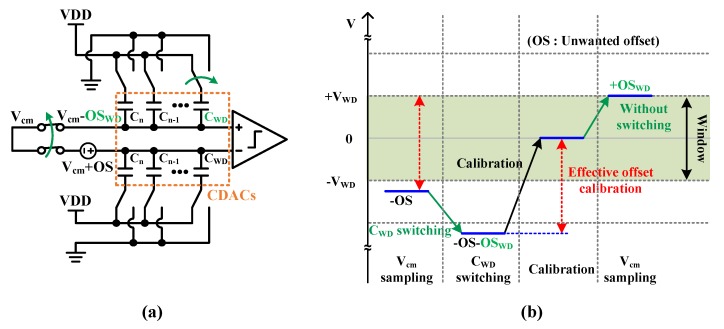
The offset calibration after C_WD_ switching to force desired offset.

In practically, because the comparator has a random offset which is unwanted offset (OS), it is necessary to force only the desired offset (OS_WD_) corresponding to the window width. For this, the differential CDACs sample the common-mode voltage (V_cm_) and the C_WD_ on the positive input side is switched from VDD to GND which shifts the sampled input difference to “-OS + (-OS_WD_)”. It is described in Figure 12a,b. This is equivalent to having offset of “-OS + (-OS_WD_)”, so if the offset calibration logic compensates it, the input difference will change to zero again. After storing the calibrated offset in the comparator, sampling the V_cm_ again, if the C_WD_ is not switched, the offset of OS_WD_ is forced as described in Figure 12b. It is because the offset calibration compensates the unwanted offset “-OS” as well as “-OS_WD_“ made by C_WD_ switching. Therefore, the only desired offset (OS_WD_) value remains.

Note that because the window width of the proposed window detector is determined by the switched capacitor (C_WD_) ratio and it has a similar structure to sub-ADCs, it is thus advantageous to resist the PVT variations than comparison time-based window detector [20, 23]. If the offset is forced on both comparators, the comparators can be used as window detector with only one conversion cycle. 

Finally, compared with previous works [20,22,23], the proposed circuit does not require the additional calibration to adjust the window width, thanks to its resistance to PVT variations. Furthermore, because it requires only one comparison cycle, eliminating the need for the extra dummy-SAR ADC to compensate for input impedance variations.

### 3.2. Timing-Skew Calibration Algorithm

The mean absolute deviation-based (MAD) timing-skew calibration [23] is adopted to reduce digital complexity and power consumption. Because MAD does not need multiplier, unlike variance-based calibration. The detail of MAD timing-skew calibration is as follows.

**Figure 13:**
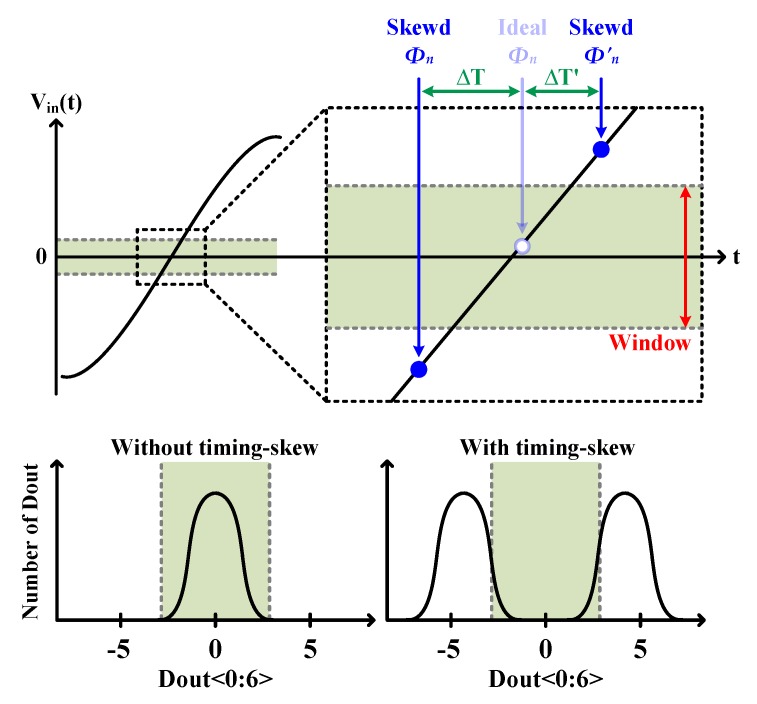
Histograms of *D_out_* without and with timing skew.

If there is no timing-skew, the digital outputs of each sub-ADC, *D_out_*’s, should be gathered near the zero because the window is set near the zero-crossing as seen in Figure 13. Therefore, the mean absolute values of *D_out_*’s, *E*(|*D_out_*|), tends to be very small [23]. However, if there is timing skew, *E*(|*D_out_*|) may become very large, because *D_out_*’s are distributed far from the zero. Therefore, the timing skew can be minimized by adjusting the sampling clock in a direction that can minimize *E*(|*D_out_*|).

**Figure 14:**
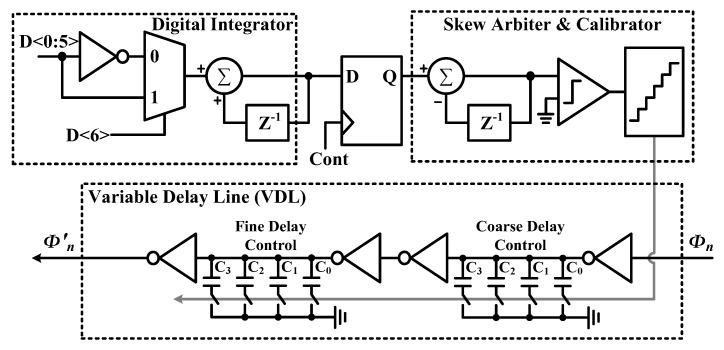
Block diagram of the proposed timing-skew calibration.

Figure 14 shows the block diagram of the proposed timing-skew calibration. The calibrator initially controls the VDL to lead or lag the sampling clock in an arbitrary direction. And then, the digital integrator takes absolute values of *D_out_*’s and integrates them for each cycle. After the integration, the timing-skew arbiter compares *E*(|*D_out_*|) with the previous one and investigates whether the timing skew is reduced or increased compared to the previous cycle. Then, if the timing skew is reduced, the arbiter commands the calibrator to keep the adjustment direction of VDL the same as before (keeping the sampling clock lead or lag as it did in the previous cycle). Other if the timing skew increases, the arbiter instructs the calibrator to invert the adjustment direction of VDL in the previous cycle.

To help readers better understand the above procedure, let us look at an example of the operation of each block in the *n^th^* cycle. After all, the *D_out_*’s at *n^th^* cycle are integrated by the digital integrator, *n^th^* mean value of |*D_out_*|'s, *E_n_*(|*D_out_*|) is compared with the previous *E_n-1_*(|*D_out_*|). If *E_n_*(|*D_out_*|) ≤ *E_n-1_*(|*D_out_*|), the arbiter judges that the calibrator is currently working correctly and instructs the calibrator to run the same as the last cycle. In the opposite case, the arbiter instructs the calibrator to operate in reverse to the previous cycle. Then the calibrator controls the VDL to make the sampling clock lead or lag, resulting in timing-skew reduction at *(n+1)^th^* cycle. Note that the timing-skew calibration operates in the background to track the voltage–temperature variations.

Meanwhile, to cover the estimated timing skew (3σ = 25 ps) acquired from the Monte-Carlo simulation and post-layout extraction, the VDL’s control range is set to ±28 ps, and the accuracy is set to ~1 ps to achieve the SNR above 40 dB at *f_in_* = 1.25 GHz. Plus, to avoid too much load on the VDL, it utilizes a coarse-fine structure, allowing the delay to be linearly controlled.

## 4. Results

**Figure 15:**
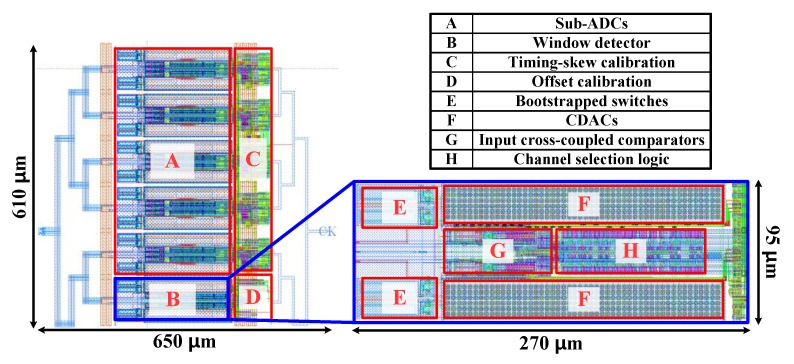
Top layout of the proposed time-interleaved SAR ADC.

To verify the proposed timing-skew calibration technique using the comparator offset-based window detector, we have performed intensive works, including designing a 7-bit, 2.5 GS/s 5-channel time-interleaved SAR ADC in 65 nm CMOS process and post-layout simulations. The top layout of the proposed time-interleaved SAR ADC is presented in Figure 15. The active area was 0.4 mm^2^, of which the calibration logics and window detector SAR ADC occupied 0.05 mm^2^ and 0.026 mm^2^, respectively. The proposed window detector only occupied 6.5% of the total area. The total power consumption of the ADC was 24 mW at 1.2 V supply. The proposed window detector only consumes 11% of the total power. The detailed SAR ADC design process and the various works using it are as follows.

In our time-interleaved SAR ADC design, we first focused on minimizing the offset and gain mismatches that cause not only window detection errors but also degrade SNR significantly. To this end, a full-custom metal-oxide-metal (MOM) capacitor was designed for a unit capacitor (~ 4fF) of CDAC to minimize the gain mismatch. Thanks to proper layout and good matching property, the gain mismatch became small enough to ignore the impact on target degradation. 

**Figure 16:**
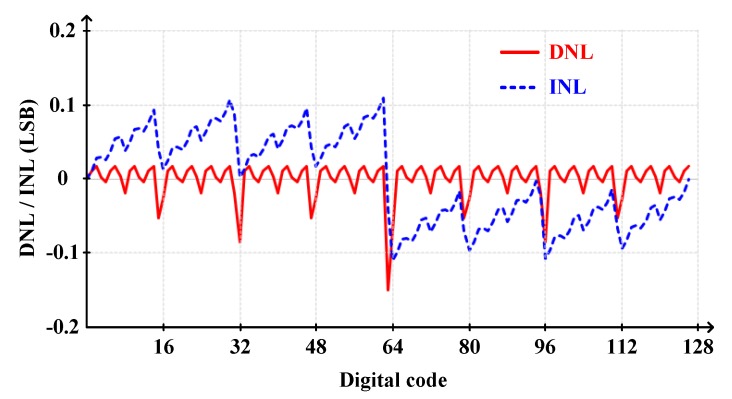
Differential non-linearity (DNL) and integral non-linearity (INL) with post-layout extraction of CDAC and an additional 1% random mismatch.

Figure 16 shows the differential non-linearity (DNL) and integral non-linearity (INL) with post-layout extraction of CDAC and an additional 1% random mismatch. As shown in simulation results (Figure 16), the DNL and INL were below 0.15 LSB.

In addition, regarding the offset mismatch, assuming that the process random mismatch represents a Gaussian distribution, the offset can be predicted by curve-fitting the probability of the comparator output "1" to the normal cumulative distribution function (CDF). The estimated offset through the Monte-Carlo simulation was ±48 mV (3σ).

**Figure 17:**
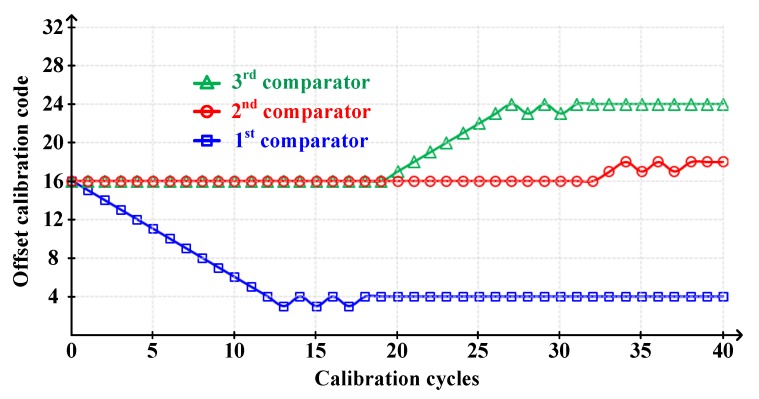
The offset calibration convergence for differential-difference comparators (DDCs).

The offset calibration convergence for DDCs is illustrated in Figure 17. As aforementioned, because single calibration logic calibrated the 17 comparators sequentially, the *n^th^* comparator must be calibrated after calibration for *(n-1)^th^*. Therefore, the calibrated comparators kept its calibration data in the register, while others that have not yet been calibrated maintain its initial data. The 5-bit calibration with ~5 mV accuracy was used for the DDCs, because they did not need coarse-fine calibration, unlike window detecting comparators. It was enough to achieve the SNR above 40 dB. The offset of each comparator was modeled by the Monte-Carlo simulation. In fact, the 15 DDCs were calibrated, but for easy readability, we only show three representative simulation results in the figure.

**Figure 18:**
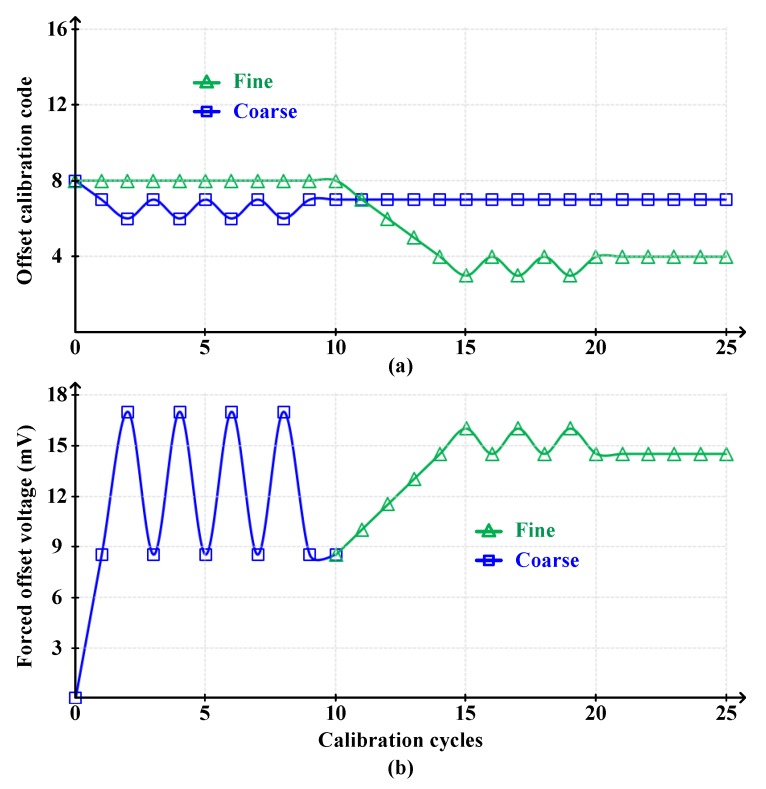
(**a**) The coarse-fine offset calibration convergence for window detecting comparator, and (**b**) its corresponding forced offset voltage.

Figure 18 shows the coarse-fine offset calibration convergence for window detecting comparator, and its corresponding forced offset voltage. To force 15 mV offset, the ~8.5 mV coarse calibration was performed first, followed by ~1.5 mV fine calibration. As the calibration progressed, the offset voltage was forced to ~15 mV corresponding to 1 LSB. Thus, the window width was set to 2 LSB (±15 mV)

**Figure 19:**
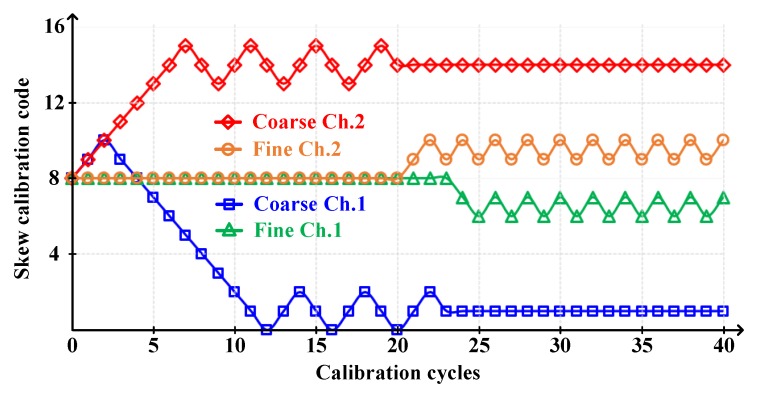
The coarse-fine timing-skew calibration convergence.

In the case of the sub-ADC, Ch.1 and Ch.2 had +23 ps and -20 ps timing skews, respectively, the timing-skew calibration convergence is described in Figure 19. The coarse calibration was performed first with ~3 ps accuracy, followed by fine calibration with ~1 ps accuracy. The total calibration range was ~±28 ps. In total, five sub-ADCs were calibrated, but due to lack of space, we only report two simulation results in this paper.

**Figure 20:**
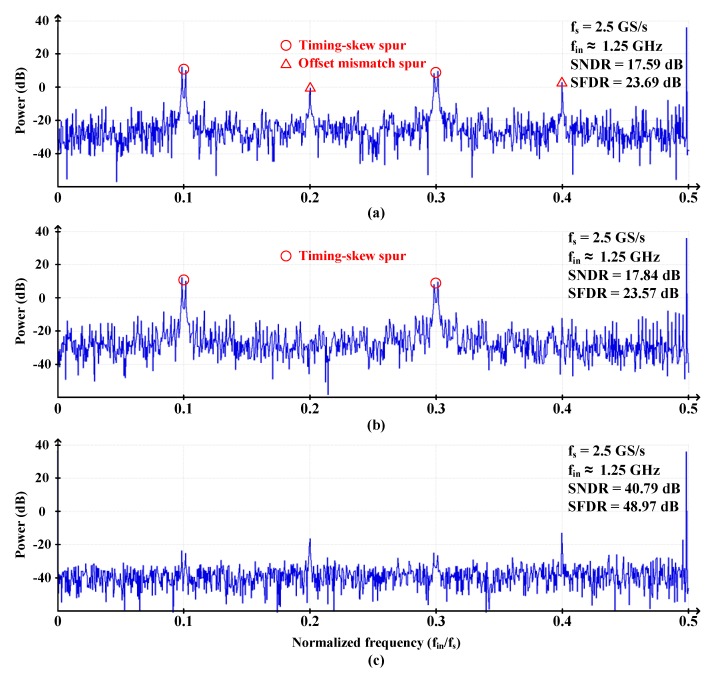
Fast Fourier transform (FFT) analysis of a sinusoidal input signal at Nyquist frequency (**a**) without calibration, (**b**) with offset calibration only, and (**c**) with both offset and timing-skew calibration (4096 points for FFT).

We performed the fast Fourier transform (FFT) analysis of a sinusoidal input signal at Nyquist frequency, and the results are shown in Figure 20. Before the calibrations, spurs caused by the offset and timing skew significantly degraded SNDR and SFDR, reaching 17.59 dB and 23.69 dB, respectively, as seen in Figure 20a.

The offset mismatch spurs were suppressed from 2.92 dB to −12.28 dB after offset calibration. However, due to the timing-skew spurs, the SNDR and SFDR were still in 17.84 dB and 23.57 dB, respectively, as shown in Figure 20b.

The timing-skew spurs were suppressed from 12.4 dB to −23.68 dB after timing-skew calibration. After performing both calibrations, even though the spurs were not completely disappeared, they were suppressed enough to achieve 40 dB SNDR. Eventually, the SNDR and SFDR at Nyquist frequency were 40.79 dB and 48.97 dB, respectively, as seen in Figure 20c.

Finally, Table 1 compares the proposed window detector with previous works. Compared to the previously published window detectors, the proposed circuit does not require additional calibration because of immunity to PVT variation and mitigates the burden of extra dummy-ADC. And the digital complexity is lower than a variance-based calibration because MAD does not use a digital multiplier. 

Table 2 summarizes and compares the performance of the proposed time-interleaved SAR ADC to previously published time-interleaved ADCs with a similar sampling rate and resolution. The proposed time-interleaved SAR ADC shows the best SNDR among the comparisons and achieves the top-flight Walden figure-of-merit (FoM_w_) of 108-fJ/conversion step at the Nyquist frequency.

## 5. Conclusions

Beginning with the thorough analysis of the advantages and disadvantages of previous time-interleaved SAR ADC designs and their indispensable timing-skew calibration techniques, we have proposed the timing-skew calibration technique using the comparator offset-based window detector. With the proposed calibration algorithm, the design methodology and operation principle of the comparator offset-based window detector are provided in detail. To demonstrate the effectiveness of the proposed technique on the timing-skew calibration, a 7-bit, 2.5 GS/s 5-channel time-interleaved SAR ADC was designed using 65 nm CMOS technology and intensive works were performed. It proves that the proposed calibration scheme well suppressed the mismatch spurs and improves the SNDR and SFDR from 17.59 dB and 23.69 dB to 40.79 dB and 48.97 dB, respectively. The calibration only increased by 6.5% effective area and 11% power consumption, which can be translated to achieving the best FoM_w_ among the comparisons.

## Figures and Tables

**Table 1:** Feature comparison.

	This Work ^1^	[20]	[22] ^1^	[23]
PVT sensitivity	robust	sensitive	robust	sensitive
Additional calibration	no	needed	no	needed
Area overhead	medium	low	high	low
Calibration method	MAD	variance	variance	MAD
Digital complexity	low	high	high	low

^1^ Post-layout simulation results.

**Table 2:** Performance comparison.

	This Work ^1^	[5]	[7]	[17]	[19]
Architecture	TI SAR	TI SAR	TI Subranging	TI SAR	TI SAR
Technology (nm)	65	45	65	40	40
Supply voltage (V)	1.2	1.1	1	1.2	1.1
Sampling rate (GSPS)	2.5	2.5	2.2	2.64	2
Resolution (bit)	7	7	7	8	8
SFDR (dB) at Nyquist	48.97	43	45.95	-	55
SNDR (dB) at Nyquist	40.79	34	37.96	38	39.4
Power (mW)	24	50	40	39	54.2
FoM_w_^2^ (fJ/conv.-step)	108	480	280	230	355

^1^ Post-layout simulation results. ^2^ FoM_w_ = power / (f_s_·2^ENOB at Nyquist^), where ENOB = (SNDR-1.76) / 6.02.
